# Landscape genetic structure of *Scirpus mariqueter *reveals a putatively adaptive differentiation under strong gene flow in estuaries

**DOI:** 10.1002/ece3.4793

**Published:** 2019-02-28

**Authors:** Mei Yang, Chengyuan Xu, Pierre Duchesne, Qiang Ma, Ganqiang Yin, Yang Fang, Fan Lu, Wenju Zhang

**Affiliations:** ^1^ College of Agriculture Yangtze University Jingzhou China; ^2^ Ministry of Education Key Laboratory for Biodiversity Science and Ecological Engineering, and Coastal Ecosystems Research Station of the Yangtze River Estuary Fudan University Shanghai China; ^3^ School of Health, Medical and Applied Sciences Central Queensland University Bundaberg Queensland Australia; ^4^ Department of Biology University Laval Québec Quebec Canada; ^5^ Shanghai Chongming Dongtan National Nature Reserve Shanghai China

**Keywords:** AFLP, colonization history, estuary, gene flow, genetic structure, isolation by distance, isolation by environment, local adaptation

## Abstract

Estuarine organisms grow in highly heterogeneous habitats, and their genetic differentiation is driven by selective and neutral processes as well as population colonization history. However, the relative importance of the processes that underlie genetic structure is still puzzling. *Scirpus mariqueter* is a perennial grass almost limited in the Changjiang River estuary and its adjacent Qiantang River estuary. Here, using amplified fragment length polymorphism (AFLP), a moderate‐high level of genetic differentiation among populations (range *F*_ST_: 0.0310–0.3325) was showed despite large ongoing dispersal. FLOCK assigned all individuals to 13 clusters and revealed a complex genetic structure. Some genetic clusters were limited in peripheries compared with very mixing constitution in center populations, suggesting local adaptation was more likely to occur in peripheral populations. 21 candidate outliers under positive selection were detected, and further, the differentiation patterns correlated with geographic distance, salinity difference, and colonization history were analyzed with or without the outliers. Combined results of AMOVA and IBD based on different dataset, it was found that the effects of geographic distance and population colonization history on isolation seemed to be promoted by divergent selection. However, none‐liner IBE pattern indicates the effects of salinity were overwhelmed by spatial distance or other ecological processes in certain areas and also suggests that salinity was not the only selective factor driving population differentiation. These results together indicate that geographic distance, salinity difference, and colonization history co‐contributed in shaping the genetic structure of *S. mariqueter* and that their relative importance was correlated with spatial scale and environment gradient.

## 1 INTRODUCTION

Gene flow and divergent selection are the two most opposite forces to determine population structure in nature (Freeland, Biss, Conrad, & Silvertown, 2010; Räsänen & Hendry, 2008; Sambatti & Rice, 2006). Gene flow may disturb the effect of divergent selection and prevent local adaptation (Garant, Kruuk, McCleery, & Sheldon, 2007; Kawecki & Ebert, 2004; Sexton, Hangartner, & Hoffmann, 2014; Slatkin, 1985), and on the contrary, divergent selection can limit gene flow through eliminating maladapted immigrants and the evolution of reproductive isolation (Cheviron & Brumfield, 2009; Quintela et al., 2014; Schluter, 2000). However, local adaptation can evolve in the presence of gene flow, and even sometimes, gene flow may promote local adaptation through the introduction of genetic variation, the spread of advantageous alleles, nonrandom dispersal, and demographic benefits (Räsänen & Hendry, 2008). In the last few years, there has been a growing interest in understanding how gene flow and divergent selection interact to generate spatial pattern of genetic variation in heterogeneous habitats (Ferchaud & Hansen, 2016; Stanton, Galen, & Shore, 1997; Tigano & Friesen, 2016), and more recently in the marine environment (Diopere et al., 2018; Rodríguez‐Zárate et al., 2018; Sexton et al., 2014). A central challenge has been presented to disentangle the relative contributions of selective and neutral processes (such as gene flow and genetic drift) underlying genetic variation (McCairns & Bernatchez, 2008; Räsänen & Hendry, 2008; Sexton et al., 2014; Tigano & Friesen, 2016).

Estuary may be the best laboratory to reveal the interaction between divergent selection and gene flow (Bible & Sanford, 2016; McCairns & Bernatchez, 2008). It represents the transitional zone between freshwater from inland and salt water from open sea (Potter, Chuwen, Hoeksema, & Elliott, 2010; Pritchard, 1967), where various processes including physical, chemical, biological, and geological dynamics are immensely complex (Wolowicz, Sokolowski, & Lasota, 2007). The distinguishing attribute of estuaries is the impact of abiotic characteristics, such as the mixing of two water sources, the rise and fall of the tides and ocean currents. This attribute makes the estuary system exceptionally variable in space and in time, especially in salinity gradients and tide levels, and creates dynamic and heterogeneous habitats (Wolowicz et al., 2007; Xin, Wang, Lu, Robinson, & Li, 2015), which may drive divergent selection. On the other hand, estuary is an open system without physical boundaries of dispersal, and the agitation of tide and freshwater connects different regions of an estuary and accelerates dispersals of all floating propagules in water, including seeds, eggs, larva, etc., which are likely to result in high gene flow among populations. It is intriguing what pattern of spatial genetic structure will be displayed in estuarine species under the contrasting effects of selection pressure and gene flow.

To date, only a few species in estuary have been studied on their genetic structure and researchers mainly focused on a small group of animals (Bible & Sanford, 2016; Bilton, Paula, & Bishop, 2002; Dennenmoser, Vamosi, Nolte, & Rogers, 2017; McCairns & Bernatchez, 2012; Sanford & Kelly, 2011) while few higher plant species have been studied (Bilton et al., 2002; but see, e.g., Ngeve, Stocken, Menemenlis, Koedam, & Triest, 2016; Ngeve, Stocken, Menemenlis, Koedam, & Triest, 2017). Additionally, these studies have shown that many are more structured than could be expected despite a lack of barriers of dispersal in estuarine system, suggesting some dispersal limitation through geographical distance, environmental variation, or other cryptic barriers promotes isolation (Kesäniemi, Hansen, Banta, & Knott, 2014). And also, the frequent finding of adaptive differentiation under high gene flow also indicates the environmental dissimilarity of estuary may play an important role in shaping genetic structure, and IBE (i.e., isolation by environment; Sexton et al., 2014; Wang & Bradburd, 2014) is common and often may contribute more genetic differentiation than IBD (i.e., isolation by distance; Wright, 1943). For example, the population genetic differentiation of estuarine species can be affected by variable oceanic currents (Ngeve et al., 2016; White et al., 2010), salinity (Gaggiotti et al., 2009; Shikano, Ramadevi, & Merila, 2010), temperature (Quintela et al. 2014; Giles, Saenz‐Agudelo, Hussey, Ravasi, & Berumen, 2015), tidal flooding (Heydel et al., 2017), and dispersal behavior (Gaggiotti et al., 2009). However, there has been complex genetic structure described as “chaotic” in marine environments when it cannot be explained or barriers to dispersal cannot be identified (Toonen and Grosberg 2011; Kesäniemi et al., 2014; Cornwell, Fisher, Morgan, & Neigel, 2016; Norderhaug et al., 2016; Miller, Baird, Oosterom, Mondon, & King, 2018), suggesting that there may be other isolation mode or cryptic limit to dispersal. For example, on a large geographic scale, the genetic pattern may be the result of historical events or past colonization history (Tahvanainen et al., 2012; Arnaud‐Haond et al. 2014; Sahyoun, Guidetti, Franco, & Planes, 2016; Maas et al., 2018), but also it may be due to complicated life history (Dennenmoser, Rogers, & Vamosi, 2014; Kesäniemi et al., 2014; Miller et al., 2018) and dispersal behavior (Becquet et al., 2013; Ngeve et al., 2017) as well as physiological or ecological limit to dispersal. In this case, because several different factors acting both spatially and temporally can lead to chaotic patterns in genetic structure, and they are frequently confounded (Maas et al., 2018); it is difficult to clear identify which factors are most important.

Here, we focus on an estuarine species, *Scirpus mariqueter* Tang & F. T. Wang (Cyperaceae), which is almost limited in the Changjiang (Yangtze) River estuary (CRE) and its adjacent Qiantang River estuary (QRE; Ou, Fang, & Shen, 1992). *S. mariqueter* grows in the lowest intertidal zone as a pioneer species forming dense meadows usually in front of *Phragmites australis* and sometimes can consist of single‐species communities covering ~100 km^2^ (Ou et al., 1992). *S. mariqueter* has been confirmed to play a key role in accelerating the development of islands and foreshores in the estuaries (Ou et al., 1992; Yang, 1998). In addition, its tubers and achenes are important food sources for several million migratory birds every year (Ma et al., 2003). However, recently, this species has been threatened by an invasive species, *Spartina alterniflora* Loisel., and also destroyed by frequent reclamation (Chen, Li, Zhong, & Chen, 2004). The reclamation events caused new populations establishing in the outside of isolating seawalls and thereby re‐isolating populations in the inside of seawalls. Possibly, colonization or reclamation process also played a role in structuring the genetic make‐up of the populations. There is a pressing need for developing protective measures, which also requires detailed information on the genetic structure of this species. It is challenging to predict genetic structure of *S. mariqueter* because so many stochastic and potentially complex processes, including selection pressure under environmental variation, dispersal ability, and reclamation process, might affect the genetic structure.

In this study, using amplified fragment length polymorphism (AFLP), we quantified the distribution of genetic variations and the migration among populations of *S. mariqueter *under heterogeneous environments of two adjacent estuaries, the CRE and the QRE. In addition, this structure may be driven by neutral and/or selective processes; thus, we look for loci potentially affected by selection (outlier loci). To disentangle the relative roles of the different evolutionary forces acting on genetic structure of this species, it is useful to combine the information on levels of gene flow obtained from neutral loci with the information from outliers that are likely to be of selective signs. In this sense, it is hypothesized that if outlier loci do not show the same IBD or IBE pattern as neutral loci, but showing a direct correlation between genetic differentiation and geographical distance or environmental variables, or colonization history, the evidence for the relative importance of above processes in shaping the structure of this species will be found.

## 2 MATERIALS AND METHODS

### 2.1 Study species and sample locations

For a long time, *S. mariqueter *has been considered as an endemic species of China, occurring in the CRE and the QRE (Ou et al., 1992). This species is a perennial clonal herb, which usually expands vegetatively by tubers with rhizome connection and reproduces sexually by seeds. *S. mariqueter* flowers from June to August. Although its flowers are wind pollinated and protogynous (see in Supporting information Figure S1), usually implying expected outcrossing, it is also highly self‐compatible (Yang et al., 2013). Achenes are matured in summer–autumn, and dispersal occurs mostly via achenes and tubers by currents and waterfowls and also by boats navigated among seaports. The discrete patches of *S. mariqueter* tend to be clear away when suffered reclamation which separate tide, but the new established patches occurred soon, and thus, the colonization history of population can be estimated from the constructed time of the latest reclamation.

**Figure 1:**
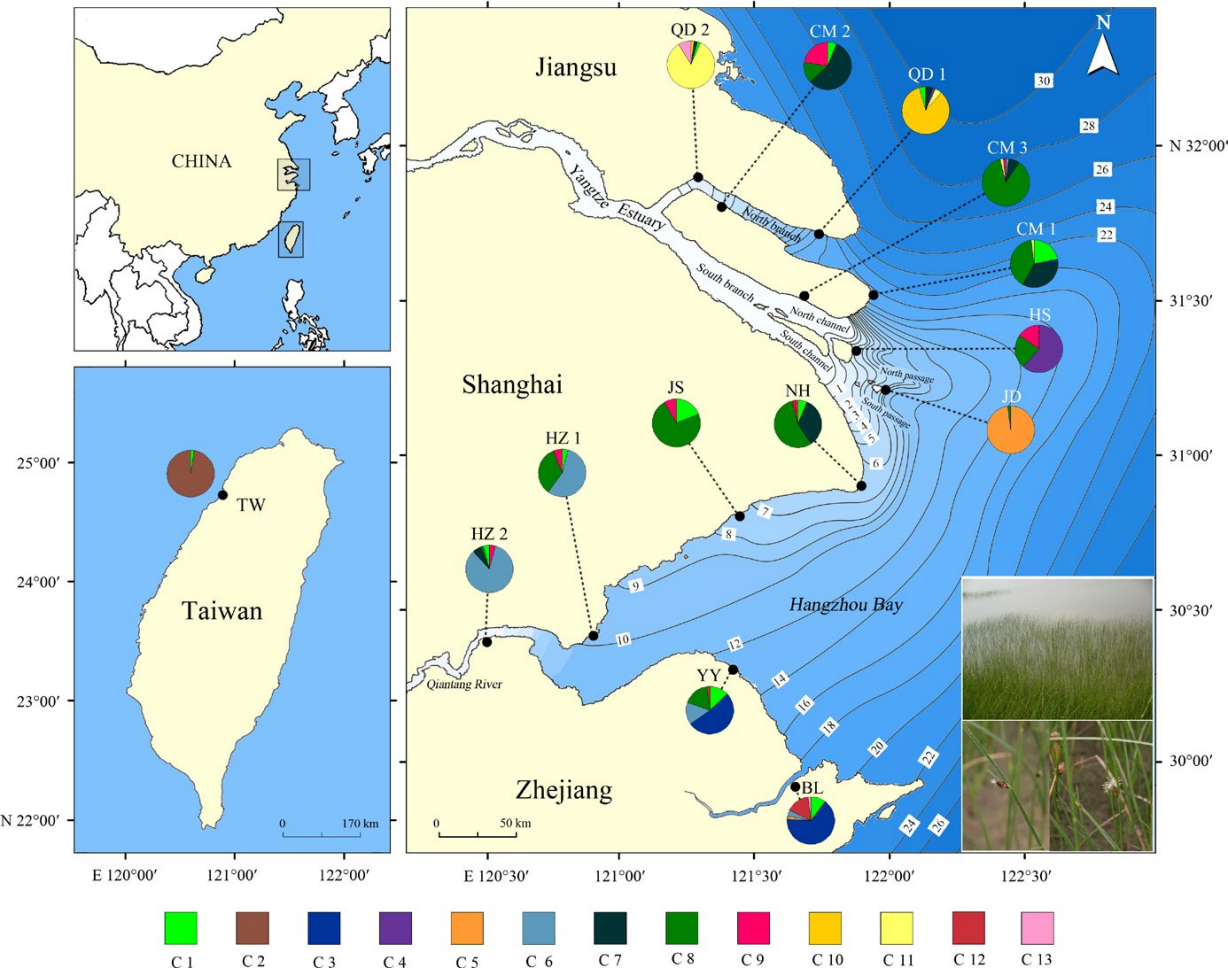
The geographic range of *Scirpus mariqueter*, the salinity level of the Changjiang River estuary (CRE) and the Qiantang River estuary (QRE), and the locations of sampled populations in this study. Photograph of *Scirpus mariqueter* from the Changjiang River Estuary (photograph credit: Mei Yang). Pie graphs show the genetic clusters and their relative proportion in different populations (the abbreviation of each cluster is corresponded to those designated in Table 4). Probability isoclines and numbers on these lines show the extent of the surface salinity (‰ or ppt) of the two estuaries in summer (redrawn according to Chen, 1988; Kong et al. 1994; Hu et al., 1995 and Bao et al., 2013). Population codes are corresponded to those designated in Table 1

The Changjiang River, is one of the largest rivers in the world, discharges a large amount of freshwater into the East China Sea through the CRE (Chen, 1988; Yang et al., 2006). The CRE has a complex structure because of the existence of a few alluvial islands. It is divided into the north branch and the south branch by Chongming Island, and the latter is again divided into the north passage and the south passage by another alluvial island, Changxing Island (Figure 1). This complex estuary has resulted in highly heterogeneous habitats with dramatically different salinity, tide, and currents (Kong, He, Ding, & Hu, 2004; Wang, Li, Zhou, & Gao, 2011; Xue et al., 2009; Zhang et al., 2013; Zheng, Ding, & Hu, 2008). Temporal and spatial variation in salinity is mainly controlled by the relative importance of river–ocean mixing. For example, only 1%–3% of freshwater discharge input to the north branch; hence, the salinity of north branch is far higher than south branch (Kong et al., 2004). Over all, the order of the salinity of these branches, from high to low, is the north branch, the south passage, and the north passage (Hu, Hu, Gu, Su, & Gu, 1995; Xue et al., 2009; Zheng et al., 2008), and within the same branch, the salinity increases from nearshore to offshore (Figure 1). In addition, at each location, the salinity changes over timescales of days (tide fluctuation), seasons (river inflow is different in wet season and dry season), and years (annual weather anomalies; Hu et al., 1995; Xue et al., 2009). These results reflect complicate temporal and spatial changes in salinity. Adjacent to the CRE, the QRE is a typical funnel‐shaped estuary with higher salinity in most regions than the CRE due to only with about 1/10 freshwater discharge of the latter and has stronger tide influence compared to the CRE (Yang, Zhu, & Zhu, 2001). These two estuaries consist of a highly connected and extremely heterogeneous system.

### 2.2 Sample and data collection

In this study, samples were collected from fourteen discrete locations along the coastal lines (Figure 1), which cover the known distributional range of *S. mariqueter*. Thirteen populations were located at the CRE and the QRE and one at the Gaomei wetlands from Jhonggang River Estuary (JRE) of Taiwan, China. Since the locations in the same region were suffered simultaneous reclamation process, and new population could be founded outside the seawall; thus, the populations from the same region have the same colonization history. According to colonization histories of these locations, these 14 populations were defined to eight groups from different geographic region: group 1 (QD1 and QD2 from Qidong), group 2 (CM1, CM2, and CM3 from Chongming Island), group 3 (NH and JS from Shanghai), group 4 (HS from Hengsha Island), group 5 (JD from Jiuduan shoal), group 6 (HZ1 and HZ2 from Hangzhou), group 7 (YY and BL from Ningbo), and group 8 (TW from Taiwan; Table 1). Approximate coastal geographical distances between population pairs were obtained with Google Earth software. Pairwise geographical distance between populations that located in the CRE and QRE ranged from 20 to 340 km, but more far from TW population (~800 km; Figure 1, Table 2). Taking into account that the growing period of *S. mariqueter* is May‐October, the estimations of average surface salinity in summer of each location were obtained from previous studies (Table 1). The absolute values of average pairwise salinity differences between sampling points were then calculated. Considering that *S. mariqueter* holds the strong ability of clonal reproduction, we sampled randomly with 10–20 m sampling intervals to avoid sampling the same clone. About 50 individuals from each population were collected except for the population of Hengsha Island (HS), where only 13 individuals were available due to very limited population size of this population. Each individual was, respectively, stored in a plastic bag and then dried with silica.

**Table 1:** Geographic locations, salinity levels, sample size, and genetic parameters for each of the 14 populations of *S. mariqueter* in China

Estuary	Group	Pop ID	Coordinates	Salinity (ppt)	*n*	PLP (%)	*H*_j _(*SE*)	*h*_s _(*SE*)
CRE	1	QD1	31°40′N, 121°41′E	9.44	44	51.6	0.1630 (0.00752)	0.1438 (0.00819)
		QD2	31°31′N, 121°58′E	0.18	46	58.1	0.1819 (0.00780)	0.1506 (0.01077)
	2	CM1	31°31′N, 121°58′E	5.00	50	55.1	0.1712 (0.00788)	0.1404 (0.00614)
		CM2	31°47′N, 121°26′E	0.27	49	54.4	0.1750 (0.00810)	0.1526 (0.00726)
		CM3	31°29′N, 121°42′E	0.14	53	50.2	0.1636 (0.00804)	0.1392 (0.00632)
	3	NH	30°51′N, 121°54′E	6.29	49	61.8	0.1912 (0.00779)	0.1571 (0.00767)
		JS	30°42′N, 121°24′E	10.46	48	54.4	0.2075 (0.00791)	0.1767 (0.00941)
	4	HS	31°21′N, 121°52′E	0.50	13	53.2	0.1811 (0.00841)	0.1771 (0.01014)
	5	JD	31°10′N, 121°58′E	1.20	50	66.8	0.1722 (0.00801)	0.1494 (0.00735)
QRE	6	HZ1	30°22′N, 120°52′E	9.30	50	61.8	0.1881 (0.00765)	0.1556 (0.00629)
		HZ2	30°16′N, 120°22′E	0.62	50	55.3	0.1630 (0.00759)	0.1312 (0.00443)
	7	YY	30°11′N, 121°31′E	11.54	46	53.2	0.1708 (0.00781)	0.1523 (0.00742)
		BL	29°56′N, 121°40′E	11.10	50	60.8	0.1944 (0.00815)	0.1659 (0.00903)
JRE	8	TW	24°18′N, 120°32′E	–	43	49.3	0.1592 (0.00786)	0.1392 (0.00596)

Note. The average value of salinity were measured in the surface water, and data were from Chen (1988), Hu et al. (1995), Kong et al. (2004), and Bao et al. (2013)

**Table 2:** Pairwise *F*_ST_ (below the diagonal) and geographical distances (km; above the diagonal) for 14 *S. mariqueter* populations

	QD2	QD1	CM1	CM2	CM3	NH	JD	HS	JS	HZ1	HZ2	YY	BL	TW
QD2		53.9	87.8	18.8	119.4	165.1	126.8	109.4	221.2	278.8	333.8	252.2	270.3	883.0
QD1	0.0729		36.2	30.5	67.2	113. 4	75.2	57.8.0	169.5	227.2	282.2	198.3	216.4.2	831. 3
CM1	0.1291	0.1278		66.0	31.6	77.2	39.0	21.6	133.3	191.0	246.0	162.1	180.2	794.6
CM2	0.1538	0.1580	0.0534		101.0	146.6	108.4	87.6	202.7	260.3	315.3	231.5.3	249.8	864.0
CM3	0.2021	0.1913	0.0799	0.1135		78.8	43.6	20.6	134.9	192.5	247.5	153.8	192.1	809.4
NH	0.1576	0.1518	0.0538	0.0740	0.0348		38.1	58.2	56.1	113.7	168.7	85.0	103.2	756.3
JD	0.1831	0.1789	0.1294	0.1206	0.1828	0.1589		22.6	94.2	151. 8	207.0	123.0	141.2	718.2
HS	0.1397	0.1925	0.1712	0.1455	0.2672	0.2019	0.1380		114.3	171.9	226.9	143.2	161.2	776.1
JS	0.1667	0.1520	0.0878	0.1146	0.0816	0.0492	0.1903	0.2054		58.8	103.8	64.0	110.0	727.0
HZ1	0.2098	0.1989	0.1019	0.0963	0.0655	0.0596	0.1666	0.2317	0.0961		55.0	56.8	127.3	734.4
HZ2	0.2789	0.2482	0.1359	0.1273	0.1232	0.1031	0.1840	0.2680	0.1703	0.0310		112.1	181.6	789.8
YY	0.2311	0.2048	0.1095	0.1422	0.1296	0.0811	0.1843	0.2275	0.1351	0.0909	0.0998		69.5	677.3
BL	0.1605	0.1623	0.1155	0.1086	0.1521	0.0948	0.1438	0.1341	0.1162	0.1117	0.1399	0.0548		660.1
TW	0.3325	0.2759	0.2048	0.2274	0.2306	0.2093	0.1465	0.3121	0.2640	0.2146	0.2111	0.2156	0.2358	

Genomic DNA was isolated from 5‐cm dried leaf tissue using CTAB (hexadecyltrimethylammonium bromide)‐based method (Doyle & Doyle 1987) from 10‐cm long‐dried leaf material. DNA quality and concentration were estimated on 1% agarose gels. We select AFLP genome scans to detect genetic structure. About 50 ng of DNA were used for AFLP analysis according to Vos et al. (1995) with minor modifications. Each individual plant was fingerprinted with five fluorescent dye‐labeled selective primer combinations: FAM‐EcoR1‐AAC/Mse1‐CTG, FAM‐EcoR1‐AAC/Mse1‐CAG, HEX‐EcoR1‐AGC/Mse1‐CTG, HEX‐EcoR1‐AAC/Mse1‐CAT, ROX‐EcoR1‐AGC/Mse1‐CAA. All PCRs were performed on ABI thermocycler 2720. To ensure reproducibility, all process maintained consistency in the duration of the study. The amplified fragments were separated by capillary electrophoresis on an ABI Prism 3730 Genetic Analyser with the internal size standard Liz 500 (Applied Biosystems). And then, AFLP markers were scored (1 as present, 0 as absent) using the GeneMapper 3.7 software (Applied Biosystems). Only unambiguous fragments were analyzed and transferred into a binary matrix. In order to reduce scoring errors, fragment peaks with fluorescence values >100 were considered as loci, and 48 samples were repeated for all processes to detect differences in allele scoring. The error rate was calculated as the proportion of fragments that could not be reproduced and the locus of error rate over 10% was discarded from the dataset. Finally, the resulting adjusted binary matrix was assembled for subsequent analysis.

### 2.3 Detection of outlier loci

AFLP genome scans can be a very useful approach to detect loci directly or linked with genome regions under selection. Genomic loci under selection (i.e., outlier loci) were investigated under the assumption that loci with uneven distribution are expected to be more genetic differentiation between populations than neutral alleles. To minimize the risk of detecting false positives, two different basic approaches were applied for detection of loci that are putatively under selection. First, we used the hierarchical Bayesian method described in Beaumont and Balding (2004) as implemented in BAYESCAN software. BAYESCAN estimates population‐specific *F*_ST_ coefficients and uses a cutoff based on the mode of the posterior distribution. The program decomposed *F*_ST_ into locus‐ and population‐specific components and was run by setting sample size to 10,000 and the thinning interval to 50 (Foll & Gaggiotti, 2008). The loci with a posterior probability over 0.99 were retained as outliers, corresponding to a Bayes Factor >2. Secondly, we used the Fdist approach by Beaumont & Nichols implemented in MCHEZA (Antao & Beaumont, 2011), which applies a multitest correction based on false discovery rate (FDR, which is the proportion of false positives among the tests found to be significant) to avoid high overestimation of the percentage of outliers (Caballero, Quesada, & Rolan‐Alvarez, 2008). Loci with an unusually high *F*_ST_ are putatively under directional selection, while loci with low *F*_ST_ value are considered to be potentially under stabilizing selection. The neutral distribution was modeled based on 500,000 data points generated through coalescent simulations under symmetric island model. The runs were conducted with the following settings: 100,000 iterations and 95%, 99%, and 99.5% CIs; loci with a significant *P*‐value at an FDR threshold of 10% were considered candidate loci; *F*_ST_ values higher than expected were considered under positive selection. After revealing selection signatures, loci were distributed in three AFLP sub datasets according to the detection pattern: the positive, neutral, and balancing datasets (see Section 3). For each sub dataset, 1,000 bootstrapped *F*_ST_ values matrices from AFLP‐SURV 1.0 (Vekemans, Beauwens, Lemaire, & Roldan‐Ruiz, 2002) were generated in order to estimate subsequently analysis of IBD, IBE, and AMOVA.

### 2.4 Genetic diversity, differentiation, and isolation by distance/environments

We characterized the overall level of genetic diversity within population, estimating: the proportion of polymorphic loci (*PPL*), Nei's gene diversity (*H*_j_), and the Bayesian estimate of gene diversity (*h*_S_). The first two calculations were carried out using AFLP‐SURV 1.0 (Vekemans et al., 2002) with nonuniform prior distribution and assuming Hardy–Weinberg genotypic proportions. AFLP‐fragment frequencies were estimated using the reliable square root method (Lynch & Milligan, 1994) and total gene diversity and average gene diversity measured at the same time. Bayesian estimate of gene diversity in each populations, *h*_s_, were calculated incorporates uncertainty about Hardy‐Weinberg proportions with HICKORY 1.0 (Holsinger, Lewis, & Dey, 2002). HICKORY uses a Bayesian estimator of population structure that builds an explicit genetic model and estimates the proportion of total genetic variability that occurs among populations. It estimates *θ* ^B^, an approximation of *F*_ST_, which does not assume Hardy–Weinberg equilibrium. The default parameters and the f‐free model, a model that does not estimate f (inbreeding within populations) were used to obtain *θ* ^B^. *θ* ^B ^statistics were calculated with different models using program Hickory 1.0: (a) a full model with noninformative priors for *f*, (b) a model in which *f = *0, (c) a model in which *θ* ^B^ = 0, and (d) a f‐free model. These models were compared using the deviance information criterion (DIC). The model with the smallest value was chosen. We set default sampling parameters: burn‐in = 50,000, sampling = 250,000, thin = 50.

In order to identify effects of colonization history on genetic structure, we used AMOVA. Total genetic diversity was partitioned among groups, among populations, and within populations by carrying out a hierarchy AMOVA on Euclidean pairwise distances among individuals using GENALEX 6.5 (Peakall & Smouse, 2012) with 999 permutations. Eight population groups were defined based on their population colonization history.

To investigate the relative importance of spatial distance and salinity difference on genetic structure, we tested for IBD and IBE based on the three datasets: all loci, positive, and neutral dataset. For IBD test, we applied traditional method of a Mantel test as implemented in GENALEX 6.5 (Peakall & Smouse, 2012). We used triangular matrix of pairwise *F*_ST_ values and triangular matrix of pairwise geographic distances as obtained earlier. In the context of local adaptation may be confounded by asymmetric dispersal among populations and by selective processes acting on the fate of immigrants, isolation by environments also been calculated to examine the association between the genetic and salinity difference. The average surface salinity in summer (peak growing season of *S. mariqueter*) was selected as an environment factor to test the effect of IBE. These correlations were performed using SPSS 15.0 software. All of above analysis were carried out with and without outlier loci to determine whether or not our results were being influenced by loci which may be under selection.

### 2.5 Genetic structure and contemporary estimates of dispersal

Assignment test is a common genetic method to provide contemporary or short‐term estimates of dispersal among populations (Campbell, Duchesne, & Bernatchez, 2003). Several different approaches were used to ascertain populations’ genetic structure. First, the AFLP binary matrix was analyzed using a Bayesian model‐based clustering method, as implemented in STRUCTURE 2.2 (Falush, Stephens, & Pritchard, 2007; Pritchard, Stephens, & Donnelly, 2000). We chose a burn‐in period of 30,000 iterations and chain length of 100,000, respectively. Independent runs with K (the number of populations) iteratively set from 2 to 14. Each run was parameterized following a model of admixture and correlated allele frequencies. After assessing the distribution of P(X|K) and Ln(K) values, all individuals were partitioned into K clusters based on the probability values. And also, the most likely number of genetic clusters (K) was detected following the approach presented by Evanno, Regnaut, and Goudet (2005).

Given that the true values of K could not be obtained to visualize structure (see in Section 3), furthermore, we used a model‐free iterative reallocation method, FLOCK (Duchesne & Turgeon, 2009, 2009, 2012) to estimate the number of populations, K. This method is robust to population inbreeding and nonzero relatedness among sampled individuals because it creates clusters based on maximizing multi‐locus genetic similarity rather than minimizing deviations from HWE and LD. In this method, samples are initially partitioned randomly into *K* clusters (*K* ≥ 2), allele frequencies are estimated for each of the K clusters, and each individual is then reallocated to the cluster that maximizes its likelihood score. Twenty repeated reallocations are performed within each run, and fifty runs are carried out for each K. Strong consistency among runs (resulting in “plateaus” of identical mean LLOD scores) is used to indicate the most likely number of clusters (Duchesne & Turgeon, 2012). Although it is not run explicitly with *K* = 1, FLOCK does test for *K* = 1. In short, *K* = 1 is the default hypothesis and is retained if no plateau of mean LLOD scores is found for any *K* ≥ 2. Next, we collected validated clusters as source populations and used AFLPOP 1.0 (Duchesne & Bernatchez, 2002) for allocation procedure. Individuals were allocated on the basis of LLOD (difference in log likelihood between the highest likelihood and the second highest likelihood), and then, the decisions were made by the simulated *P* value.

## 3 RESULTS

### 3.1 Detection of loci under selection

**Figure 2:**
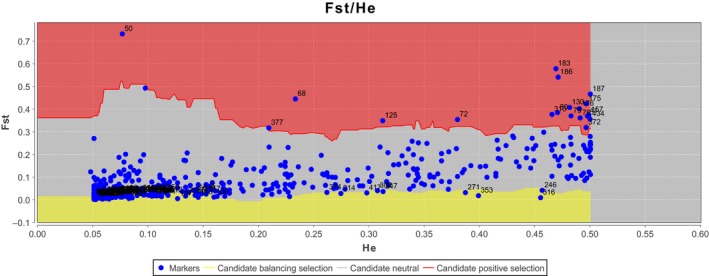
Plot of *F*_ST_ values against heterozygosity estimates generated with MCHEZA. Each point corresponds to an AFLP locus (*N* = 434). The three lines represent, respectively, the 1%, 50% (median) and 99% percentiles of the simulated distribution of neutral expectations based on 10^5^ realizations

A total of 641 samples were scored for 434 loci, and these loci were retained for further statistical analyses. BAYESCAN detected 81 loci under selection at a *q*‐value threshold of 0.10. Of them, 60 were detected in only one single analysis and thus considered as false positives (i.e., loci detected because of the 5% type I error). MCHEZA detected only 21 loci (Figure 2) which also could be detected in the former programs. We considered these 21 loci as reliable outliers under selection because they are common in the two programs and further formed “positive dataset.” In total, 11 loci were attached to population QD1 and QD2, 3 to TW, 3 to JD and TW, 1 to population HS and JS, 1 to HZ1, HZ2, 1 to YY and BL, and 1 to CM1, CM2, CM3 in particular (Supporting information Table S1). No loci were attached to population NH, which is located on the confluence of CRE and QRE. Additionally, MCHEZA detected 97 loci with balancing selection and the remaining 316 neutral loci were formed “neutral dataset.”.

### 3.2 Genetic diversity and among‐population differentiation

The proportion of polymorphic loci (*PPL*) ranged from 49.3% to 66.8% (Table 1), with a mean value of 56.1%. Total genetic diversity and average gene diversity measured over all loci and all populations were moderate (*H*_t_ = 0.2101, *H*_w_ = 0.1773). Both estimates of gene diversity varied only a little among populations. The range of estimates was from 0.1592 to 0.2075 assuming H–W equilibrium (Table 1), and similar but slightly lower value (see *h*_S_ in Table 1) were obtained with the Bayesian approach, which was not constrained by assumptions of H–W equilibrium.

The pairwise differentiation (*F*_ST_) between populations varied widely, ranging from 0.0310 (HZ1–HZ2) to 0.3325 (QD2‐TW), and all *F*_ST_ values were significantly different from zero (*p* < 0.001) (Table 2). The analysis gave us almost similar mean estimate of *θ* ^B^ whether we using the full model (0.3870 ± 0.009) or *f‐*free model (0.3554 ± 0.019). This was higher than the traditional estimate of the overall *F*_ST_ between all populations (*F*_ST_ = 0.1565 ± 0.106) when assuming H–W genotypic proportions or AMOVA estimate (*F*_ST_ = 0.1857). Comparison of the deviance information criterion (DIC) in different models, the highest DIC value was attained with a model of no structure (*θ* ^B^ = 0) provided further evidence that there is relatively high level of population genetic differences (Jacquemyn, Honnay, Looy, & Breyne, 2006).

An AMOVA revealed different levels of genetic structuring for *S. mariqueter* populations. The hierarchical AMOVA on 641 samples based on all loci revealed that 80.51% of the variation within populations, while 6.85% was due to variation between populations and 12.64% was due to variation between groups. When based on 21 outliers, the hierarchical AMOVA revealed that 47.19% of the variation within populations, while 9.21% was due to variation between populations and relatively high proportion (43.60%) was due to variation between groups with different population colonization history (Table 3).

**Table 3:** Results of AMOVA analysis of AFLP data with and without outlier loci

Source of variation	*df*	Sum of squares		Variance components		Percentage of variation	
		All loci	outliers	All loci	outliers	All loci	outliers
Among groups	7	5,186.682	1,093.588	3.933	1.788	12.64	43.60
Among populations	6	1,364.408	122.176	3.757	0.378	6.85	9.21
Within populations	627	27,687.244	1,213.263	44.158	1.935	80.51	47.19
Total	640	34,238.334	2,429.027	54.848	4.101	100	100

Note. Groups cluster to different colonization history of each population (Table 1).

### 3.3 Genetic structure and ongoing gene flow

**Figure 3:**
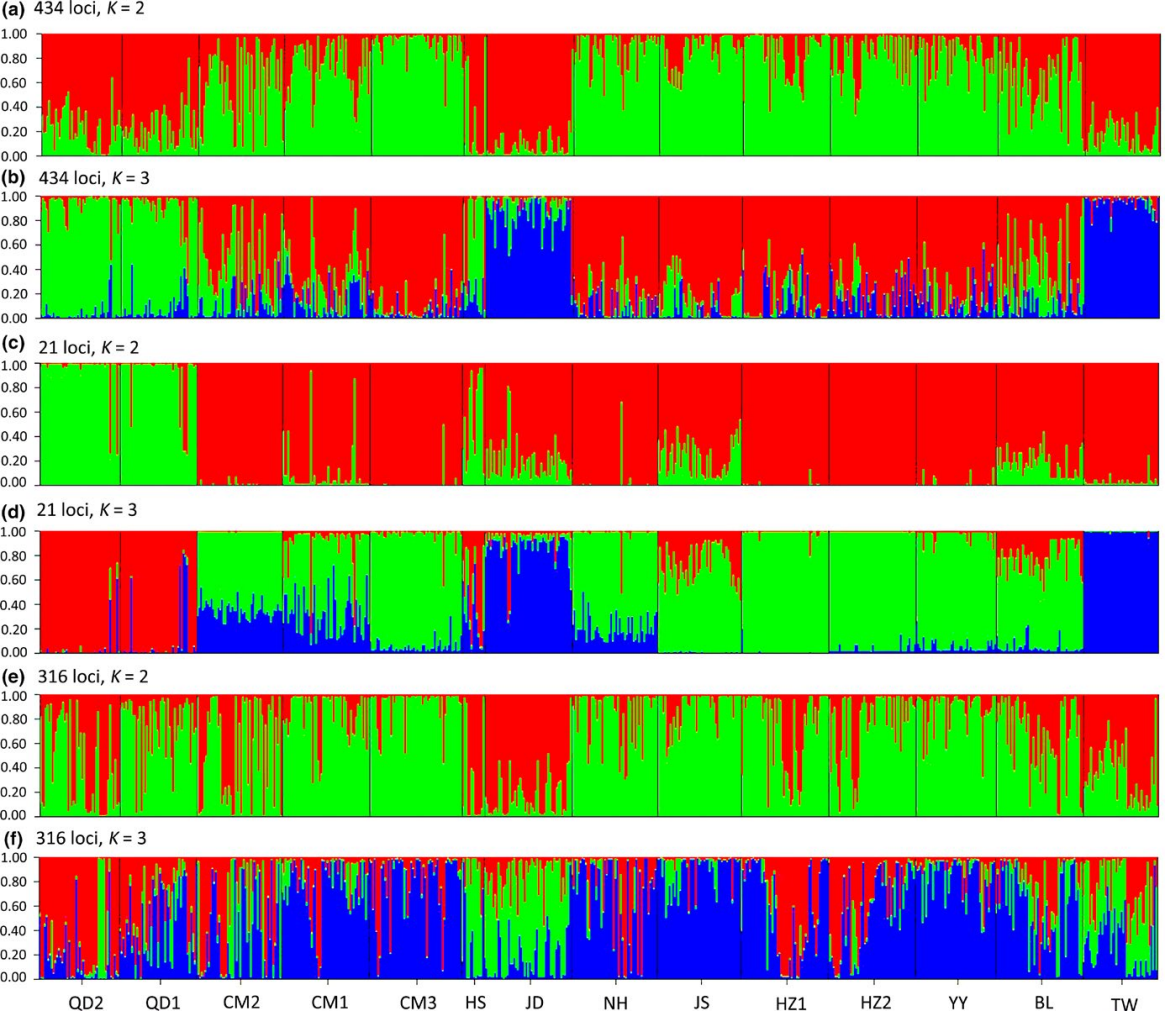
Bayesian clustering for 14 populations of *S. mariqueter* STRUCTURE. Each individual is represented by a vertical bar divided into two or three segments corresponding to its membership coefficients in the two (*K* = 2) or three (*K* = 3) inferred clusters. Each tonality represents a different cluster and black lines separate the individuals of different localities. (a) and (b), analyses using the entire genetic dataset of 434 loci with *K* = 2 and *K* = 3, respectively; (c) and (d), analyses considering outlier dataset of 21 loci with *K* = 2 and *K* = 3, respectively; (e) and (f), analyses taking into account only the 316 neutral loci with *K* = 2 and *K* = 3, respectively. The *K* value is determined from the mean estimated Δ*K* (Evanno et al, 2005). More information found in Supporting information Figure S2

The Bayesian clustering method based on STRUCTURE could not infer an optimal structuring into *K* populations: Ln (*K*) kept increasing with increasing *K*. Separate STRUCTURE analysis including all individuals for each dataset after applying posterior Δ*K* statistic (Evanno et al., 2005), however, likewise resulted in a most likely number of *K* = 2 (based on all loci or neutral dataset) or *K* = 3 (based on outlier dataset; Supporting information Figure S2). When *K* = 2, no distinct groups were found based on all loci or neutral dataset (Figure 3a,e), while two distinct clusters corresponding to “QD1, QD2” and the other populations based on outlier dataset, (Figure 3c). When *K* = 3, based on all loci and outlier datasets, we found three distinct clusters corresponding to “QD1, QD2,” “JD, TW,” and the other populations, respectively (Figure 3b,d). However, no distinct clusters were found based on neutral loci and all individual were much admixed (Figure 3f).

FLOCK analysis, the non‐Bayesian approach, however, found a partition into 13 clusters (i.e., genetic types, abbreviated as “C”) as the most likely solution (Table 4). According to the assignment test by FLOCK, each individual was assigned to one of thirteen clusters. About one‐quarter of all individuals (167, 26.05%) were assigned into a single cluster (C8) while three clusters (C4, C12, and C13) only included a few individuals (<2%), respectively. Other individuals (450, 70.20%) were assigned to the remaining 9 clusters (Table 4). There were six narrowly distributed clusters, and they almost occurred, respectively, in only one population, including C2, C4, C5, C10, C12, and C13. Other clusters were widespread and composed of individuals from several different populations, especially for C1, C7, C8, and C9.

**Table 4:** Assignment numbers and allocation of *S. mariqueter* individuals (*n* = 641) statistics from 14 locations

Allocated to	QD1	QD2	CM1	CM2	CM3	HS	JD	NH	JS	HZ1	HZ2	YY	BL	TW	Allocation statistics (%)
C1	2	1	11	3	0	0	0	3	9	2	2	6	5	1	7.02
C2	0	0	0	0	0	0	0	0	0	0	0	0	0	42	6.56
C3	0	0	1	0	0	0	0	0	0	0	0	24	33	0	9.04
C4	1	0	0	0	0	8	0	0	0	0	0	0	0	0	1.40
C5	0	0	0	0	0	0	49	0	0	0	0	0	1	0	7.80
C6	0	0	0	0	1	0	0	0	0	28	42	7	2	0	12.48
C7	0	1	17	28	4	0	0	17	0	0	3	0	0	0	10.92
C8	2	0	20	7	46	3	1	27	35	17	1	8	0	0	26.05
C9	0	0	0	11	1	2	0	1	4	3	2	0	0	0	3.74
C10	37	1	0	0	0	0	0	0	0	0	0	0	0	0	5.93
C11	2	39	1	0	1	0	0	0	0	0	0	0	0	0	6.71
C12	0	0	0	0	0	0	0	1	0	0	0	1	8	0	1.56
C13	0	4	0	0	0	0	0	0	0	0	0	0	1	0	0.78

These 14 populations had different degrees of admixture and different genetic components (Figure 1 and Table 4). The most individuals of the populations QD1, QD2, JD, HZ2, BL, and TW were allocated to their origin of location. Conversely, the other populations were composite which means that these populations had high level of mixing. Besides, the majority individuals of some neighboring populations shared the same genetic clusters, such as HZ1 and HZ2, YY and BL (Figure 1). Up to 145 individuals from five central populations (CM1, CM3, NH, JS, HZ1), which were located at the central region of the study area, belonged to the same genetic type (C8). However, some close populations had very different dominant clusters and large genetic difference, such as CM2 and QD2, JD and HS. Compared with a large number of migrants between adjacent populations or populations in the central region, few individuals of some cluster were also allocated to distant populations, that is, one individual of CM1 was assigned to C3, but this cluster was mainly comprised of individuals from BL and YY, and one individual of TW was unallocated to its sampling location, but assigned to genetic type (C1) with other individuals from the CRE and the QRE.

### 3.4 Relative importance of IBD and IBE

**Figure 4:**
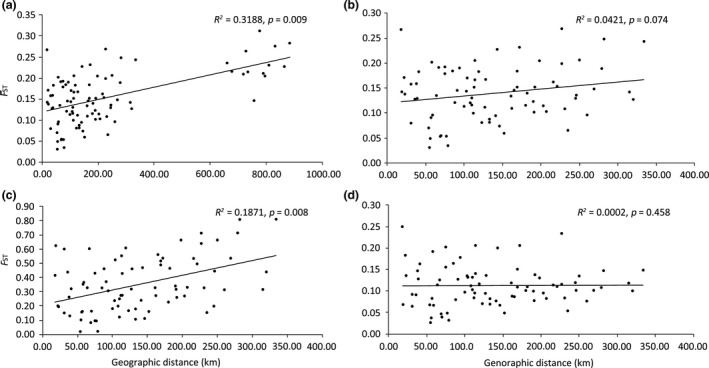
IBD analyses for all 14 population (a) and 13 populations in CRE and QRE when excluding TW population considering different dataset: all loci dataset (b), outlier loci dataset (c), and neutral dataset (d). The mantel test scatter plot shows the relationship between the pairwise genetic differentiation (*F*_ST_) and the geographic distance (km) between populations

IBD effect was revealed by the association between genetic differentiation (*F*_ST_) and geographic distance. A significantly positive relationship was found (*r* ^2^ = 0.3118, *p* = 0.009) for all 14 populations (Figure 4a). And also, when the population (TW) from Taiwan was excluded, nonsignificant results were found (*r* ^2^ = 0.0421, *p* = 0.074) based on all loci (Figure 4b). The lack of positive correlation indicates that samples are not spatially genetically structured and that isolation by distance does not play a role solely. However, for these 13 populations located in the CRE and the QRE, a significant positive relationship was detected (*r* ^2^ = 0.1871, *p* = 0.008) with 21 outliers (Figure 4c), while nonsignificant relationship with neutral dataset (Figure 4d).

**Figure 5:**
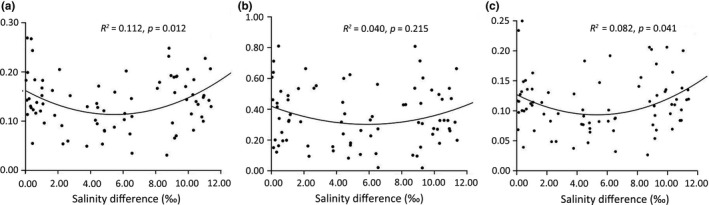
IBE analyses for 13 populations in CRE and QRE when excluding TW population considering different dataset: all loci dataset (a), outlier loci dataset (b), and neutral dataset (c). The mantel test scatter plot shows the relationship between the pairwise genetic differentiation (*F*_ST_) and the ecological distance between their locations. The ecological distance is measured by the salinity difference between sample locations, and the average surface salinity in the growing season (summer) of *S. mariquete*r was used (showed in Table 1)

IBE tests with different datasets were conducted by comparing the relationship between pairwise *F*_ST_ values and pairwise salinity differences among populations located in the CRE and the QRE (TW is excluded for lacking salinity data). A concave curve was obtained with all loci (Figure 5a): for the populations with a low salinity difference (≤4.0 ppt), a significantly negative correlation was found (*r* = −0.44, *p* = 0.015), but for those with a high salinity difference (>4.0 ppt), the correlation was significantly positive (*r* = 0.33, *p* = 0.021). When selecting the geographic distance as the controlling factor, similar results were obtained (when salinity difference ≤4.0 ppt, *r* = −0.38, *p* = 0.042; when salinity difference >4.0 ppt, *r* = 0.29, *p* = 0.047). However, no significantly positive relationship was found as expected when only considering outlier dataset, but also a concave curve was obtained (Figure 5 b): nonsignificantly correlations either with a low salinity difference (≤4.0 ppt) (*r* = −0.034, *p* = 0.300) or with a high salinity difference (>4.0 ppt) (*r* = 0.147, *p* = 0.320). In contrast, based on neutral dataset, none‐liner pattern but significantly negative correlation was found with a low salinity difference (≤4.0 ppt) (*r* = −0.358, *p* = 0.044) and a significantly positive correlation with a high salinity difference (>4.0 ppt) (*r* = 0.28, *p* = 0.05; Figure 5c).

## 4 DISCUSSION

In this work, non‐Bayesian clustering analysis using FLOCK assigned all individuals to thirteen genetic clusters and revealed a complex genetic structure of *S. mariqueter*: some clusters were limited in marginal locations compared with very mixing constitution in central populations. In another analysis, only two genetic clusters were found in STRUCTURE following Bayesian clustering method, suggesting high connectivity among populations, and both populations and individuals were varying degrees of admixture. However, a relatively high genetic differentiation value (*F*_ST_ = 0.1857) among populations was found although all locations hydrologic linked and indeed amount of migrants were detected. Considering these results, population genetic structure and its correlates with spatial distance, salinity difference and population colonization history were analyzed. The outlier loci analyses suggested that some of the AFLP loci were putatively under divergent selection, and the analyses of population genetics considering only these outliers revealed a very different pattern of differentiation from results obtained with the entire dataset or neutral dataset according to STRUCTURE, AMOVA, IBD, and IBE tests. The results suggest that the ecological forces, including environmental factors like surface salinity, spatial distance, and colonization history all play important role in shaping population structure of *S. mariqueter* and the effects may be compound.

### 4.1 Migration, gene flow and genetic structure

Every specific trait of estuarine habitats, especially the rise and fall of the tides and the exchange between saltwater and freshwater, make the spatially isolated species in estuary to be interconnected to varying degrees through dispersals of seeds or/and other propagules (Potter et al., 2010; Uncles & Stephens, 2011). For *S. mariqueter*, beyond the transport by water flow, the long‐distance dispersal mostly via achenes and vegetative propagules can also be done by many birds (Ma et al., 2003) and the boats between harbors or seaports in the CRE and the QRE. Thus, a large number of migrants among populations of *S. mariqueter* should be expected. Indeed, the assignment test showed that there was a high level of migrations among some populations. Many genetic types were shared by several populations, especially C1 and C8, which were both found in 11 populations, indicating that dispersals occurred among almost all populations, including long‐distance dispersal (one individual of TW was assigned to C1). But migrations often happened more frequently among many neighboring populations, for example, HZ1 and HZ2, BL and YY, NH and JS (Figure 1).

*S. mariqueter* is a cross‐pollination species (Ou et al., 1992; Yang et al., 2013) and has a narrow adaptive belt at its each distributed site; thus, it is reasonable for us to assume that these migrations are likely to result in gene flow among populations, which tends to reduce genetic differentiation or homogenize populations and may swamp adaptation to local conditions (Ellstrand, 2014). However, the assignment test by FLOCK showed that the gene flow among populations had been inhibited at certain degrees. Some genetic types were almost limited in one population, for example, C4, C5, and C10, suggesting these clusters were locally adapted, and gene flow was limited between populations even on small spatial distance. Especially, JD population is not far from other populations; however, only one individual from other populations was found. This observation indicates some barrier to gene flow although under strong dispersal potential. Gene flow follows migration, but not necessarily, and can only occur after successful establishments of migrants and reproduction (Tigano & Friesen, 2016). Heterogeneous habitats cannot only limit dispersal of seeds or other propagules by mis‐adaptation, but also may inhibit gene flow by natural selection (Sexton et al., 2014). The environment of both the CRE and the QRE is highly heterogeneous not only at different sites of each estuary in salinity, tide, sediment charge, etc., but also at different altimetric positions of the same intertidal zone (Bu, 2013). Thus, we propose that the heterogeneity of environments at different scales limit successful establishment of migrants among some populations and also among clusters within population, and therefore constrain gene flow between different environments, which will result in a relatively high genetic differentiation.

*F*_ST_ values support this suggestion. In our study, a relatively high genetic differentiation value (*F*_ST_ = 0.1857) was detected although all locations hydrologic linked. The greatest interregional differentiation was found between TW and QD1 population (*F*_ST_ = 0.3325), which was responded to the very long distance between them (~800 km), but when TW was excluded, the overall *F*_ST_ was 0.1263, and also exhibited a relatively high level of genetic differentiation even in the absence of physical barriers at this regional scale. Population differentiation represents a historic gene flow rather than current value (Ellstrand, 2014; Ouborg, Piquot, & Groenendael, 1999). In this vein, the coexistence of a relatively high genetic differentiation and frequent ongoing dispersal suggests that some processes, for example, selective pressure and genetic drift may restrict successful establishment of dispersal via seeds or tubers, thus reduce effective gene flow and also increase the probability of local adaptation which contribute to differentiation (Bolnick & Otto, 2013; Wang & Bradburd, 2014).

Some results give the evidence for inference of local adaptation. The outlier loci analyses detected 21 positively selected loci that potentially under selection. In these outliers, over half of selected loci are attached to QD1 and QD2, 3 to TW, and 3 to JD and TW, indicating these populations are likely to have obtained local adaptations. These populations are all at the edge of the distribution. We also noticed that the specific genetic clusters (C2, C10, C11) were only limited in these populations while there was hardly positively selected loci attached to the central populations of *S. mariqueter*. Empirical studies have shown that most geographical peripheries are also ecologically marginal (Abeli, Gentili, Mondoni, Orsenigo, & Rossi, 2014), meaning more disadvantageous environmental conditions and relatively less migrants or/and gene flow. In the periphery of a range, rather than in central populations, due to higher pressure from genetic drift, reduced effective population sizes, found effects, and restricted gene flow (Dennenmoser, Nolte, Vamosi, & Rogers, 2013; Pandey & Rajora, 2012; De Ryck et al., 2016; Sexton et al., 2014), it tends to create genetic distinct clusters and promote genetic differentiation between populations. Besides, analysis of population structure in STRUCTURE considering only outliers showed a stronger differentiation pattern compared to those obtained with the entire dataset or neutral dataset, and the most differentiation was occurred between the peripheries (QD1, QD2) and central populations. These results co‐contribute to the conclusion that local adaptation is an important driven force for genetic distinct clusters in peripheral populations.

### 4.2 Compounded effects of geographic distance, salinity difference, and colonization history for genetic differentiation

Many reported patterns of differentiation in estuarine populations have revealed a strong genetic structure and restrictions to gene flow (Dennenmoser et al., 2014; Ferchaud & Hansen, 2016; Kelly & Palumbi, 2010; McCairns & Bernatchez, 2008; De Ryck et al., 2016). This doubtless reflects barriers to dispersal, such as geographic distance and divergent selective regimes (Heydel et al., 2017; Neiva, Pearson, Valero, & Serrao, 2012), but also, in some case, population colonization history. The most common mode of isolations is IBD and IBE. The former results in gene drift, which is controlled by mutation and gene flow, and the latter causes different natural selections due to habitat heterogeneity. IBD effect was significant at large spatial scale, such as interregional scale (near 800 km between the two estuaries and Taiwan coastal areas) but nonsignificant at regional scale (approximate 20–300 km) when TW was excluded based on entire dataset. This result indicated that the *S. mariqueter* populations at the CRE or the QRE were deviate from the migration–drift equilibrium, which may be disrupted by the divergent selection process (Bradburd, Ralph, & Coop, 2013; Sexton et al., 2014; Shikano, Jarvinen, Marjamaki, Kahilainen, & Merila, 2015), the long‐distance dispersal or stochastic colonization. However, for these thirteen populations, compared with nonsignificant relationship between genetic differentiation and geographic distance with entire or neutral dataset, a significant strong IBD effect was detected with 21 outlier dataset, reflecting the effect of spatial distance on isolation seems to be promoted by divergent selection. Our findings are consistent with Jones et al. (2013), based on the AFLP analysis; under weak selection, the strength of IBD was lower than under strong selection as a result of that strong IBD can confound landscape.

When natural selection mainly drives genetic differentiation, the correlation between genetic differentiation and some environment factors often can be found (Sexton et al., 2014; Wang & Bradburd, 2014). If isolation by environment plays a key role, we expect environmentally similar locations will also be genetically similar. However, in this study, the genetic differentiation and the difference of one important environment factor, salinity, did not show a significant line‐relationship without (mantel test, *r* = 0.018, *p* = 0.379) or with outlier dataset (mantel test, *r* = −0.049, *p* = 0.374), but showed a concave curve (*r* ^2^ = 0.11, *p* = 0.012 and *r* ^2^ = 0.04, *p* = 0.215, respectively). However, this result does not mean the habitat heterogeneity of *S. mariqueter* cannot result in the genetic differentiation of this species, because a significantly positive correlation with the salinity difference >4.0 ppt (*r* = 0.33, *p* = 0.021) was found. However, we cannot explain why the genetic differentiation was significantly and negatively correlated with the salinity difference when the latter was equal to or less than 4.0 ppt (*r* = −0.44, *p* = 0.015). A potential reason is that when the salinity difference was low, the effect of natural selection on the genetic differentiation might be diluted by other factors, such as gene flow and phenotypic plasticity (the ability to tolerate salt stress under lower salinity). Some population pairs (e.g., QD2 and HZ2, QD1 and YY) with similar salinity (lower salinity difference) but were located at different estuaries, respectively, also existed a high level of genetic differentiation (Table 2). In contrast, some neighbor populations (e.g., QD1 and QD2, HZ1 and HZ2, YY and BL) had a lower genetic differentiation though with a larger salinity difference. These results indicate the effects of salinity are overwhelmed by spatial distance or other ecological process in certain areas and also suggest that salinity is not the only selective factor driving population differentiation. Only with a large salinity difference, its effect on genetic differentiation can be observed.

Genetic structure in *S. mariqueter* also could reflect “isolation by population history” which has been evidenced by recently studies (Maas et al., 2018). This mode of isolation emphasize the importance of colonizers in shaping subsequent population genetic structure, also termed “historical priority effects” (Maas et al., 2018; De Meester, Vanoverbeke, Kilsdonk, & Urban, 2016) or “historical contingency” (Fukami, Mordecai, & Ostling, 2016; Orsini, Vanoverbeke, Swillen, Mergeay, & Meester, 2013) It is meaning that the early colonizers tend to be more locally adapted in comparison with late arrivers, due to density‐dependent and evolution‐mediated dominance of early genotypes. For example, there is not a complete barrier in the open sea, as the water/ocean currents can carry propagules among estuarine populations. Such characteristics indicate that individuals who founded the population could originate from multiple source populations rather than from a single source population. However, the successful colonizers are almost the first colonizers due to density‐dependent ecological priority effects which may further mediated by evolution via adaptation to local conditions (Maas et al., 2018). This observation has been evidenced, and the high plasticity would benefit colonization of new locations. Given that *S. mariqueter* populations were suffered repeated reclamation/colonization in certain areas, past colonization history could be a major factor influencing its population genetic structure. The hierarchical AMOVA revealed that the proportion of variation among eight groups with different colonization history was very low with all loci dataset, but the value arises to 43.60% considering only outliers. This result suggests that the selected loci are closely related with past colonization history; thus, the population differentiation scenario involving the colonization history is reinforced. For example, Hengsha Island (HS) and Jiuduan shoal (JD) are alluvial islands in the CRE only with a short history (~160 years for the former and <100 years for the latter, Yang, 1998; Hu, Cheng, Hu, & Hu , 2004). These two alluvial islands are very close, but their hydrologic conditions are distinctively different from each other (e.g., salinity in Figure 1). Thus, according to the result that C5 was limited at Jiuduan shoal, we have reason to believe C5 is an adaptive genetic type to Jiuduan shoal. This fact indicates that migrants from other populations have undergone a rapid adaptive evolution at this new alluvial island. We speculate that the similar evolutionary events are likely to happen at Hengsha Island before, an elder alluvial island, since C4 was also limited at Hengsha Island. In such cases, early differentiation among sympatric colonization history populations could have been initiated through a reduction in gene flow among locally adapted groups occupying discrete environments. And also, the genetic clusters C10 and C11 were limited at QD1 and QD2, respectively, providing an explicit evidence that local adaptation is more likely to occur in peripheral populations, most likely as a consequence of historical arrival of founders with subsequent inbreeding and dispersal limitation due to the heterogeneous environment in combination with genetic drift effects.

To date, the patterns of genetic structure of estuarine populations with molecular markers cover a wide range of possible outcomes among and within species. Such observations may reflect varying degrees of isolation driven by selective and neutral processes. In this study, though *S. mariqueter* has a small distribution range with no geographical barrier, a relatively high level of genetic differentiation was found among populations of this species, and this differentiation was proved to be affected by the interaction between geographic distance and environmental variability, as well as population colonization history. The results may help to disentangle the relative contributions of the underlying processes in the formation of genetic structure among estuarine populations, especially for high plants. Besides, this study provides a signal of local adaptation occurred in peripheral populations although high migration in these estuaries, suggesting that effective conservation of *S. mariqueter* should include maintaining all populations cover its distribution range regardless of population size, thus promoting preservation.

## CONFLICT OF INTEREST

The authors declare no conflict of interests.

## AUTHOR CONTRIBUTIONS

M. Y. and W. Z. conceived and designed the research; M. Y., W. Z., F. L., and Q. M. collected samples; M. Y. and Y. F. performed the experiments; M. Y., P. D., W. Z., C. X., and G. Y. analyzed the data; M. Y. and W. Z. wrote the manuscript.

## Data Availability

Dryad Data Repository https://doi.org/10.5061/dryad.25kg375. Available data consists of "ece‐Sm‐AFLP data" including 0–1 matrix of entire loci, outliers, and neutral dataset in different sheets for all individuals, "ece‐Sm‐Mcheza" including the list of potential loci under selection with MCHEZA, DFDIST, and BAYESCAN software, respectively, and "ece‐Sm‐summary of FLOCK" including the assignment test results from FLOCK analysis.
