# Supplementary material for: Landscape genetic structure of Scirpus mariqueter reveals a putatively adaptive differentiation under strong gene flow in estuaries
Source: Ecol Evol. 2019 Feb 28;9(6):3059–74. doi: 10.1002/ece3.4793 (PMC6434575; doi:10.1002/ece3.4793)
Supplement: Supplementary file 1 [file ECE3-9-3059-s001.doc]

**Supporting Information:**

**Table S1** Candidate loci to be under directional selection detected in the *S. mariqueter* populations, obtained with MCHEZA method.

| Attached populations | Loci name |
| --- | --- |
| QD1, QD2 | 72, 76, 79, 80, 130, 157, 175, 176, 183, 186, 187 |
| JD, TW | 68, 372, 434 |
| TW | 49, 50, 177 |
| YY, BL | 377 |
| HZ1, HZ2 | 125 |
| HS, JS | 41 |
| CM1, CM2, CM3 | 310 |

**Fig. S1** Photograph of *Scirpus mariqueter* from the Changjiang River Estuary (photograph credit: Mei Yang).


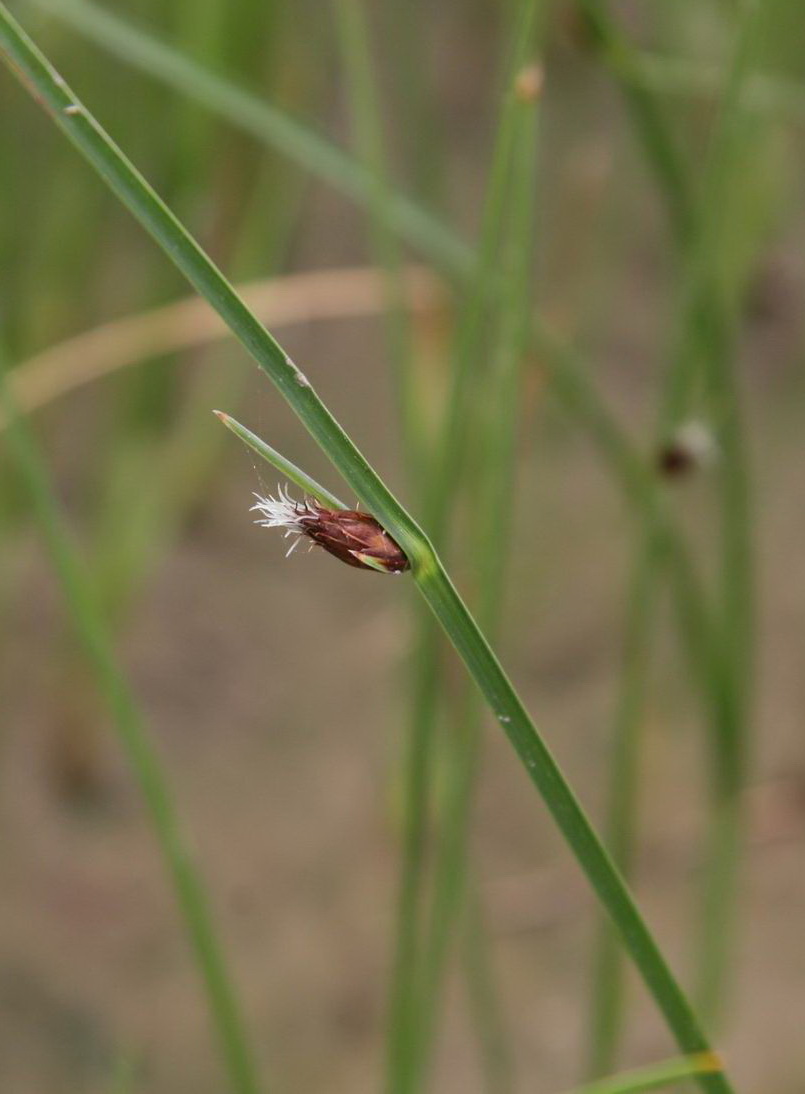

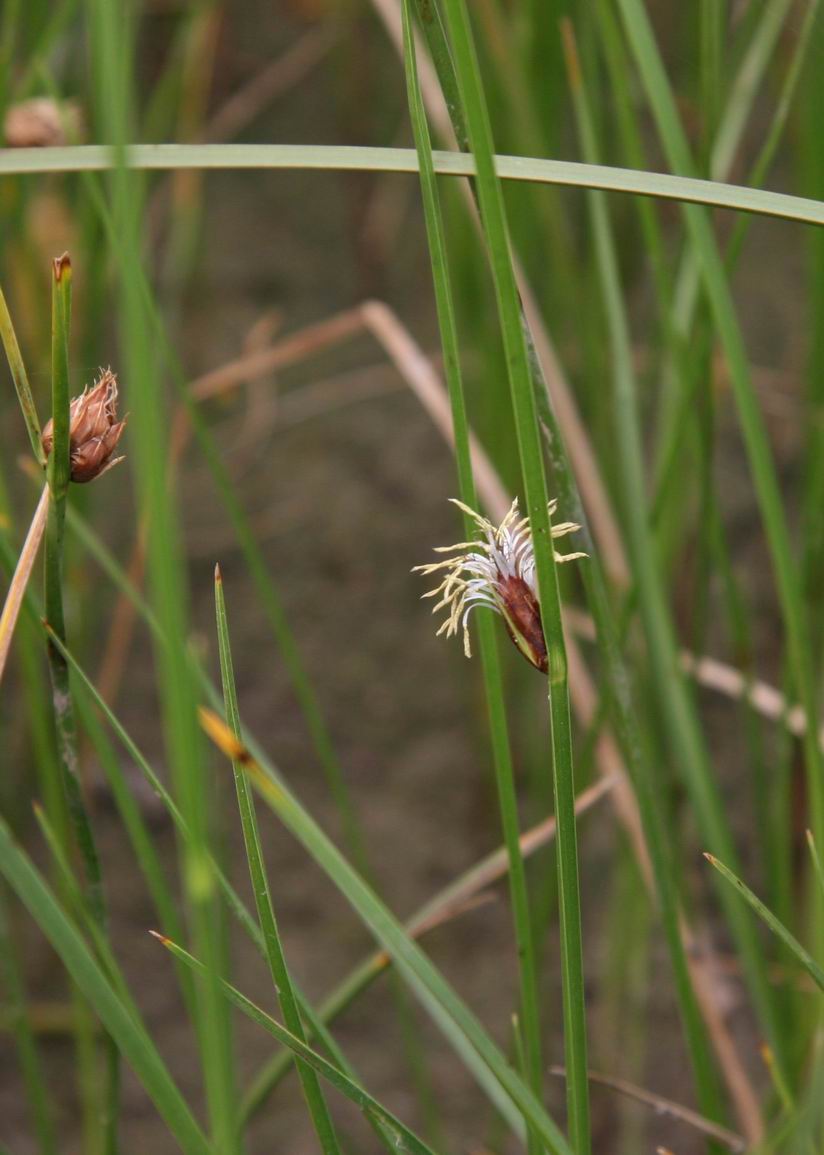


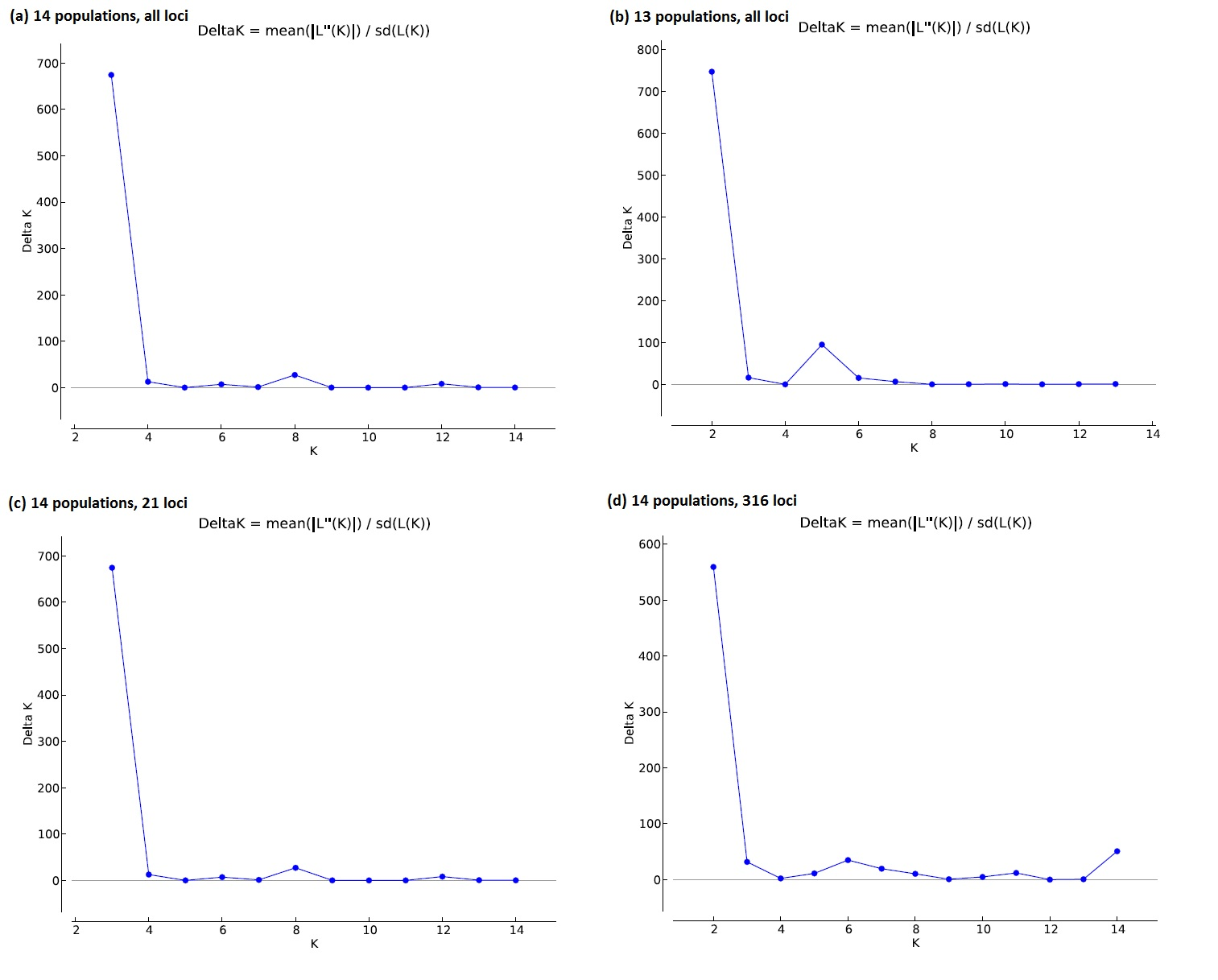
**Fig. S2** STRUCTURE Harvester results indicate the number of K using Evanno’s delta K are the most probable number of theoretical clusters. (a) and (b), K = 3 and K = 2 when using the entire dataset of 434 loci for all populations and 13 populations (excluding TW), respectively. (c), K = 3 considering outlier dataset of 21 loci. (d), K = 3 when using the 316 neutral loci.
